# The role of duel hydrolysis of soybean on functional properties and protein digestibility: a sustainable approach

**DOI:** 10.3389/fnut.2024.1444329

**Published:** 2024-09-18

**Authors:** Nishithkumar Jogi, Somya Adusumilli, Madhukar Nagesh, Sudheer Kumar Yannam, Bangera Sheshappa Mamatha

**Affiliations:** ^1^NITTE (Deemed to be University), Department of Food Safety and Nutrition, Nitte University Centre for Science Education and Research (NUCSER), Mangalore, Karnataka, India; ^2^Department of Traditional Foods and Applied Nutrition, Central Food Technological Research Institute (CFTRI), Mysore, Karnataka, India; ^3^Freeze Drying and Animal Product Technology Division, Defence Food Research Laboratory, DRDO, Ministry of Defence, Mysore, Karnataka, India

**Keywords:** soybean, duel hydrolysis, modification, functional food, bitternes

## Abstract

**Background:**

Protein hydrolysates derived from food sources contains enormous number of peptides which are composed of amino acid possessess various bioactive properties. However, the use of protein hydrolysates as a nutraceutical is hindered due to their unpleasant flavour. The study aims to enhance the biological activity and palatability of protein hydrolysates.

**Methodology:**

In the present study, soybean protein hydrolysate (SPH) was prepared using alcalase for 4 h (control). Modification of hydrolysis (MPH) was carried out by reiterating the hydrolysis of the supernatant obtained after 2 h of hydrolysis using an enzyme to 50% of alcalase during each successive hydrolysis. Samples were characterised by their physio-chemical and functional properties. Furthermore, the effect of modification on the protein digestibility and bitterness intensity using e-tongue was studied. The suppressive effect on retrogradation of corn starch was analysed using texture profile analysis.

**Results:**

The results demonstrated increased protein content by 1.6 and 1.9% in MPH compared to SPH and UNH, respectively. MPH showed 1.5- and 1.6-fold higher DH% than SPH before and after gastrointestinal digestion (*p* < 0.05). A decrease in molecular weight was found in the order of UNH > SPH > MPH. Nevertheless, MPH displayed significantly higher functional properties (*p* ≤ 0.05). The hardness of retrograded corn starch was significantly reduced in the MPH (1.21N) than SPH (1.55 N) and UNH (1.81N) compared to control (1.71N) during 7-day storage at 4°C *(p* ≤ 0.05). E-tongue analysis of MPH showed a 4-fold reduction in bitterness than SPH.

**Conclusion:**

Modification of hydrolysis of soybean has demonstrated its significance in improved DH% functional properties and palatability. In addition, improved protein digestibility with promising benefits in deferral action on retrogradation of starch over the traditional process of hydrolysis was observed. The outcome of this study contributes to the potential utilisation of MPH as an ingredient in the formulation of nutraceutical products.

## 1 Introduction

Soybean (*Glycine max*) is a good source of protein, dietary fibre, vitamins, and minerals. It has various industrial applications including extraction of edible oil or used as bio-diesels and bio-plastic materials. Protein hydrolysates are the fractions of protein that are formed during the hydrolysis of protein. These protein hydrolysates have proven to have bioactive (antioxidant, anticancer, antihypertensive, antimicrobials, etc.) and functional properties [water holding, oil holding, foam forming, emulsification, etc.), which are commonly used in animal feed and have limited use in food formulations due to low palatability (1–4). Numerous studies have been carried out on the preparation of soybean protein hydrolysates and their bioactive properties (5–7)]. The major challenge to using it as a nutraceutical food is the formation of bitter flavour during hydrolysis. Typically, hydrophobic amino acids such as glycine, alanine, valine, leucine, isoleucine, proline, phenylalanine, methionine, and tryptophan are known to contribute to bitterness in protein hydrolysates (8). Nevertheless, the bitterness of protein hydrolysates is also associated with the degree of hydrolysis (DH%), molecular weight, position of proline residues, type of enzymes used, and amino acid sequence in a peptide (9). Despite its high biological value, these problems hinder the usage of protein hydrolysates as a major constituent in a product (7, 8, 10, 11). Researchers and food industries have continued to work on improving the taste, odour, and overall acceptability of protein hydrolysates and alternative protein sources, and production methods are being explored to address sustainability and cost concerns (7, 8, 10). Thus, implementing the novel approach to produce the protein hydrolysates with the minimum process is an area of interest. Studies have been conducted on protein hydrolysates used as food additives in a high-starch gluten-free diet to improve its nutritional composition (12, 13). In our study, protein hydrolysates from soybeans were prepared by modifying the conventional enzymatic hydrolysis and studied for their characteristic features and functional properties. In addition, the incorporation of MPH into high-calorie-based corn starch is to examine its suitability in deferment of retrogradation in gelatinised corn starch. High-starch products often face severe retrogradation during cold storage which extremely affects the quality and shortens the shelf life of the products. There are studies demonstrating a combination of rice starch with ionic (xanthan gum) or non-ionic (guar gum) hydrocolloids exhibits a significant way to minimise the side effect of starch retrogradation (14, 15). Therefore, the present study intends to modify the conventional hydrolysis process to enhance the sensory qualities, functional properties, and digestibility of soybean protein hydrolysates.

## 2 Materials and methods

### 2.1 Materials

Soybean (*Glycine max)* was obtained from the local market in Mangaluru, Karnataka, India.

### 2.2 Reagents

Proteolytic enzymes such as alcalase (*Bacillus licheniformis*) (2.972 U/mL activity) were purchased from Sigma-Aldrich. Sodium hydroxide, solvents, digestive enzymes, and other chemicals used in the study were of analytical grade.

## 3 Methodology

### 3.1 Preparation of soybean protein hydrolysate (SPH)

Soybean protein hydrolysates were prepared according to Yathisha et al. (16). In brief, 500 g of dried soybean (14.01% moisture content) was soaked in potable water for 20 min followed by homogenisation with 1,000 mL of water. Inactivation of endogenous enzymes was carried out by heating the mixture at 90°C for 20 min. The homogenate was allowed to cool to 55°C, and the pH was adjusted to 8.5. Hydrolysis was carried out with 2% (w/v) alcalase for 4 h. The process was terminated at 90°C for 20 min followed by rapid cooling (4°C). The supernatant was separated and filtered after centrifuging at 10,000 rpm for 20 min. The filtrate was lyophilised and stored at −20°C for further analysis.

### 3.2 Modification of soybean protein hydrolysates (MPHs)

**Figure 1:**
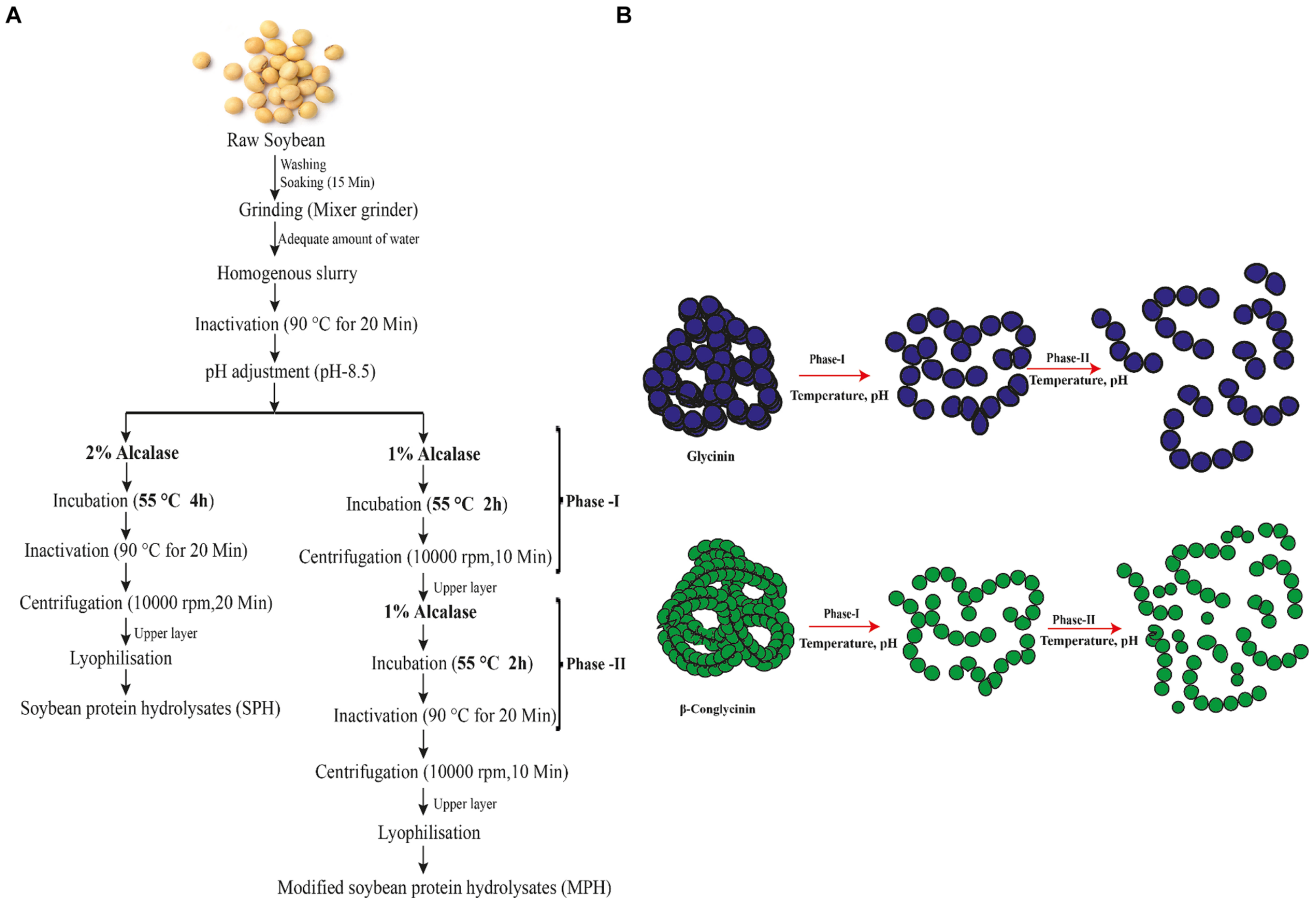
Preparation of soybean protein hydrolysates (SPHs) and modified protein hydrolysates (MPHs). **(A)** Enzymatic hydrolysis of protein from soybean is prepared by using alcalase for 4 h. In the conventional method, hydrolysis was performed for 4 h with 2% alcalase to produce soybean protein hydrolysis (SPH) which serves as a control. Modified hydrolysis of soybean (MPH) was performed by splitting the hydrolysis time and enzyme concentration into Phase-I and Phase-II. **(B)** Possible mechanism of generation of peptides during the preparation of modified protein hydrolysates (MPHs). During the hydrolysis of soybean, the main constituents of soya protein (glycinin and β-conglycinin) unfold at first and break down into peptides with varied chain lengths by the action of alcalase on hydrolysis. In Phase-I, the large trimeric and hexameric structure unfolds and forms polypeptides with varied MW. In Phase-II, the polypeptides formed in Phase-I are hydrolysed further which break down the polypeptides into smaller MW peptides.

Modification of preparation of the protein hydrolysates was carried out by splitting the hydrolysis time and enzyme concentration by 50%. The process of the preparation of SPH (control) and MPH (Modified) is given in Figure 1A. MPH was prepared by adding 1% of alcalase enzyme (v/w) to the homogenate (pH 8.5) and incubating at 55°C for 2 h followed by centrifuging at 10,000 rpm for 20 min (Phase-I). The process was repeated for 2 h with the remaining 1% of alcalase (Phase-II). The supernatant was separated and filtered after centrifuging at 10,000 rpm for 20 min. The filtrate was lyophilised and stored at −20°C for further analysis.

### 3.3 Degree of hydrolysis (DH%)

The DH% was measured by estimating the soluble nitrogen using trichloroacetic acid (TCA). In brief, protein hydrolysates (5 mg/mL) and 50 μL of 10% TCA were added and incubated at room temperature for 15 min followed by centrifugation at 5000 rpm for 10 min. The supernatant was estimated for the protein content (16). The degree of hydrolysis was calculated using the following formula:

$$\begin{array}{l} \mathrm{Degree}\;\mathrm{of}\;\mathrm{hydrolysis}\;\left(DH\%\right)\\ \;=\left(10\%TCA\;\mathrm{soluble}\;\mathrm{protein}/\mathrm{Total}\;\mathrm{protein}\right)\times 100\text{.} \end{array}$$

### 3.4 Characterization of soybean protein hydrolysates

#### 3.4.1 Molecular weight distribution by SDS-PAGE

Distribution of MW in protein hydrolysates was carried out according to the method followed by Idowu et al. (17), with slight modifications. A stacking gel of 5% and a running gel composition of 20% were used to run the electrophoresis. In brief, 50 mg/mL of protein hydrolysates were prepared in phosphate buffer and mixed well. The solution was then centrifuged at 8500 *g* for 15 min at 4°C using a cold centrifuge. The supernatant was mixed with sample buffer (0.125 M Tris–HCl, pH 6.8 containing 4% SDS with β-mercaptoethanol) and 20% glycerol at a ratio of 1:1 (v/v). Then, 30 μL of the sample was loaded onto the gel. The electrophoresis was initially run at 60 MV for 30 min followed by 90 MV for 60 min. Furthermore, the gels were fixed and stained with 0.05% (w/v), Coomassive brilliant blue R-250 in 15% methanol and 5% acetic acid and de-stained using 30% methanol and 10% acetic acid. The low-range molecular mass markers (3 to 197 kDa) were used to identify the MW of the hydrolysates.

#### 3.4.2 Amino acid composition by LC–MS

The amino acid content in SPH and MPH was estimated according to Vashishth et al. (18), with slight modifications. In brief, 100 mg of UNH, SPH, and MPH were treated with 1 mL of 6 N HCl in a separate 25 mL flask and incubated at 60°C for 24 h. Furthermore, the sample was diluted with 20 mL of HPLC grade water. A known amount of aliquot was dried and dissolved in 1 mL of 0.1 N HCl. The sample was derivatised using 5, 1, and 1 μL of borate buffer, o-phthalaldehyde (OPA), and sample, respectively. Then, 8 μL of FMOC (9-fluronitrilemethylchloroformate) reagent was added to the above mixture. The derivatised sample was analysed using an HPLC system (Waters RP-HPLC system Model-1525) with a C18 column (250 mm X 4.6 mm, no. OHSO1521) and equipped with a photodiode-array detector (PDA). The mobile phases A (2.72 g of sodium acetate trihydrate and 180 μL trimethylamine, pH 7.2 in 1000 mL water) and B [water, methanol, and acetonitrile (20:40:40) with 2.72 g sodium acetate trihydrate] were used at a flow rate of 0.45–1.0 mL/min at 338 nm for 30 min.

#### 3.4.3 Particle size distribution

The particle size of the samples was determined using the laser diffraction particle size analyser (SALD-2101 SHIMADZU, Japan) equipped with Microtrac particle size analysis software (19).

#### 3.4.4 Water activity

The water activity (a_w_) of the sample was measured using the water activity meter (Aqualab Pawkit Meter Food, USA). In brief, 0.5 g of the sample was placed on the plates and inserted into the system (19).

#### 3.4.5 Color measurements

A colour measuring system (Model CM3500D, Minolta spectrophotometer, Minolta Co., Ltd., Japan) was used to determine the Chroma and Hue of the samples. The results were expressed in L*, a*, and b* values (20).

### 3.5 Evaluation of bitterness intensity by e-tongue analysis

**Figure 2:**
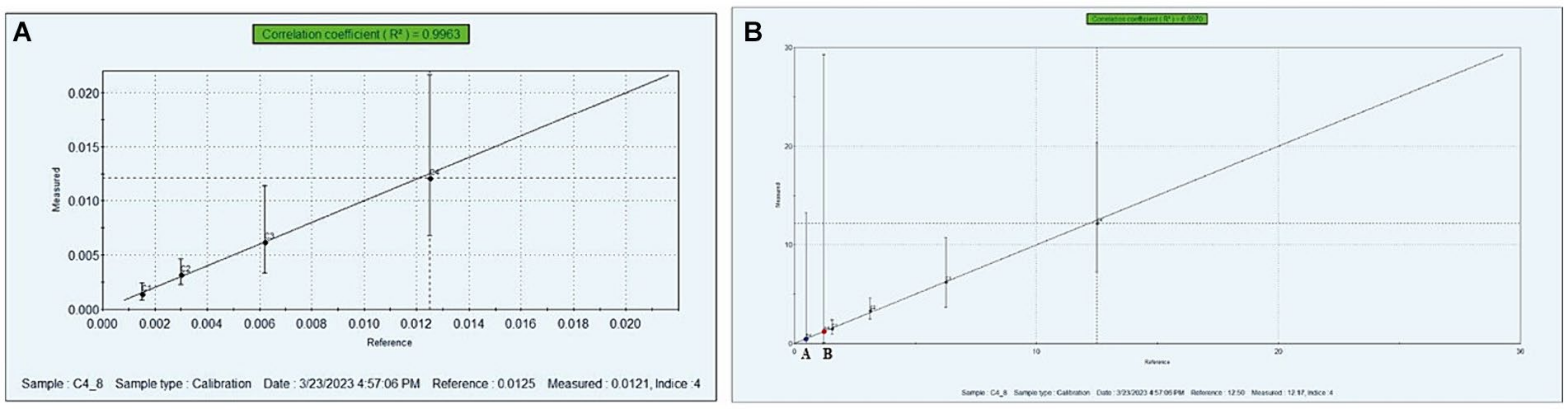
Calibration curve of standard caffeine obtained by e-tongue analysis **(A)**, bitterness intensity of SPH and MPH **(B)**. **(A)** Modified soybean protein hydrolysate (MPH), **(B)** soybean protein hydrolysate (SPH).

The bitterness evaluation of protein hydrolysates was carried out using an Electronic-Tongue (Auto-sampler, Model: ASTREE, αM.O.S Toulouse, France) as explained by Singh et al. (21). In brief, 250 mg of SPH and MPH were dissolved in 25 mL of distilled water, vortexed until fully dissolved, and then filtered through Whatman NO.1 filter paper for analysis. Different concentrations of standard caffeine (0–0.020 M) were used as a standard. The standard curve was plotted against bitterness measured values v/s reference (Figure 2A) for the bitterness in the samples.

### 3.6 Functional properties

#### 3.6.1 Solubility

The solubility of SPH and MPH was estimated at varied ranges of pH (2, 4, 6, 8, 10, and 12) as explained by Yathisha et al. (16). Next, a 200-mg sample was dissolved in 10 mL of distilled water. The pH was adjusted using 1 N HCl and 1 N NaOH. The mixture was stirred continuously at 37°C for 30 min and then centrifuged at 8000 rpm for 10 min. The supernatant was estimated for soluble proteins using Lowry’s method and calculated using the following equation.

$$\mathrm{Solubility}\;\left(\%\right)\;=\left(\mathrm{Protein content in supernatant}/\mathrm{Total protein content in the sample}\right)\times 100\text{.}$$

#### 3.6.2 Oil-holding capacity (OHC)

The OHC of SPH and MPH was determined according to Leni et al. (22). In brief, 100 mg of samples were taken in a pre-weighed vial, and 1 mL of sunflower oil was added. The samples were vortexed for 1 min and incubated for 30 min at room temperature. The mixture was centrifuged at 13,600 *g* at 25°C for 10 min (Eppendorf Germany 5810R). The final weight was taken after decanting the oil upside down (45° angle), and the OHC capacity was calculated as follows:

$${OHC}_{\left(g\;\mathrm{of oil}/g\;\mathrm{of sample}\right)}\;=\left(\mathrm{Final weight}-\mathrm{initial weight}/\mathrm{weight of the sample}\right)\text{.}$$

#### 3.6.3 Hardness of retrograded corn starch using TPA

The mixture of 10 g of corn starch in 100 mL of deionised water and 1 g of samples and guar gum was heated at 95°C for 30 min in a shaking water bath. The starch was then allowed to cool for 1 h. The gelatinised samples were prepared into a rectangle shape (2.7 cm 170 × 2.7 cm × 2 cm) and stored at 4°C. Each sample was compressed thrice using a TA-XT plus texture analyser (TA Instruments, USA) equipped with a cylindrical probe (36 mm diameter) at 1 mm/s, and the deformation level was 30% of 173 the original sample height (13).

#### 3.6.4 *In vitro* gastrointestinal (GI) digestion

The effect of GI digestion on the protein digestibility of samples was performed according to the method given by Sanjukta et al. (23), with slight modifications. The GI digestion was carried out initially using 4% pepsin (2 h) at pH 2 followed by 4% pancreatin (4 h) at pH 7.5 at 37°C. The enzyme activity was stopped by heating at 90°C for 10 min. The digesta was centrifuged and lyophilised to estimate the DH%.

### 3.7 Safety evaluation of SPH and MPH (*in vitro* and *in vivo*)

*In vitro* cell proliferation was measured by using RAW264.7 macrophage cell lines as described by Saisavoey et al. (24), with slight modifications. In brief, 1 × 10^4^ cells were seeded in 96-well plates and incubated for 48 h. The cells are then treated with SPH and MPH at different concentrations (20–100 μg) for 48 h. Then, 5 μL of 50 μg/mL of MTT was added to each well and incubated for 4 h. The supernatant in each well was removed, and 100 μL of DMSO (dimethyl sulphoxide) was added. Absorbance was read at 595 nm using a multiplate reader, and cell viability was calculated using the following formula:

$$\mathrm{Cell}\;\mathrm{death}\;\left(\%\right)=\left(A_{\mathrm{control}}-A_{\mathrm{sample}}\right)/\left(A_{\mathrm{control}}\right)\times 100\text{.}$$

*In vivo* toxicity of MPH was evaluated using male Wistar rats approved by the Institutional Animal Ethics Committee (NGSMIPS/IAEC/JUNE-2020/207). The acute toxicity of MPH was assessed as per the guidelines (25). Six male Wistar rats (150–170 kg B.W) were divided into two groups (*N* = 3) caged separately and provided adequate diet and potable water with a 12-h day and night cycle. Before the study began, the animals were fasted overnight from food, and the test group was dosed orally with 2000 mg/kg BW of MPH, whereas the control group received the same amount of distilled water. Each rat was observed carefully for 14 days for any behavioural changes and lethality. After behavioural studies, the animals were sacrificed, and vital organs (heart, kidney, lungs, and liver) were collected and examined for histopathology (26).

### 3.8 Statistical analysis

All data represent the mean value ± SD of three independent measurements. The comparison between the two groups was carried out using two-way and one-way ANOVA. GraphPad Prism software version 8.3.0 was used to analyse statistical significance at a *p*-value of ≤0.05.

## 4 Results

### 4.1 Modified method for preparation of protein hydrolysis (MPH)

Process modification of preparation of protein hydrolysate was carried out to decrease the MW and reduce the bitterness in the sample. MPH was prepared by hydrolysing with alcalase to obtain SPH with higher nutritional and functional properties. The modification of traditional protein hydrolysate preparation avails the already hydrolysed proteins into further breakdown to release shorter chain length peptides with medium or low molecular weight peptides in it. The preparation of MPH and the possible mechanism for the generation of protein hydrolysates with lower MW peptides are shown in Figure 1.

MPH showed significantly higher protein content (68.86%) compared to SPH (58.36%) and UNH (34.62%). Nevertheless, modification resulted in reduced fat content in MPH to 10.65% from SPH which had fat content of 22.41%. These results demonstrate that a decrease in the fat content and an increase in the protein content can increase the biological activity of MPH along with stability in terms of long-term storage.

### 4.2 Degree of hydrolysis (DH%)

DH of the protein hydrolysates determines the extent of breaking down of the parent protein by the proteolytic enzymes. The DH% of all the samples is tabulated in Table 1. It was observed that the modification of soybean protein hydrolysates significantly increased the DH% than the control and unhydrolysed soybean protein (*p* < 0.05). MPH showed 3.3-fold higher than UNH and 1.5-fold higher than SPH. These results demonstrate that modification could increase the DH of the protein hydrolysates which intend to exhibit various biological and functional properties of the hydrolysates.

**Table 1:** Physio-chemical and functional properties of UNH, SPH, and MPH.

Sl. No	Samples	Degree of hydrolysis (DH)	Water activity (a_w_)	Oil-holding capacity (g/g)	Color			Particle size distribution				
					L*	A*	B*	0–100 (μm)	100–300 (μm)	300–500 (μm)	500–700 (μm)	700–1,000 (μm)
1	UNH	16.52 ± 1.31^a^	0.11 ± 0.06^a^	4.747 ± 0.09^a^	77.95 ± 0.08^a^	−0.51 ± 0.03^a^	13.11 ± 0.05^a^	17.31	34.68	17.93	13.79	10.24
2	SPH	28.41 ± 0.51^b^	0.12 ± 0.06^a^	3.349 ± 0.04^b^	73.83 ± 0.77^b^	−0.65 ± 0.21^a^	19.40 ± 0.43^b^	20.81	27.9	32.19	10.6	5.67
3	MPH	43.00 ± 0.5^c^	0.09 ± 0.004^b^	3.278 ± 0.09^b^	72.99 ± 0.15^c^	−0.58 ± 0.01^a^	20.56 ± 0.03^c^	18.62	23.46	16.32	9.72	11.82

Values are represented as mean ± SD of triplicates. The difference in the alphabets among the rows and superscript indicates statistical significance (*p* ≤ 0.05) within the columns of the same groups determined using two-way ANOVA. UNH, unhydrolysed soybean protein hydrolysate; SPH, soybean protein hydrolysate; MPH, modified protein hydrolysate.

### 4.3 Molecular weight distribution by SDS-PAGE

**Figure 3:**
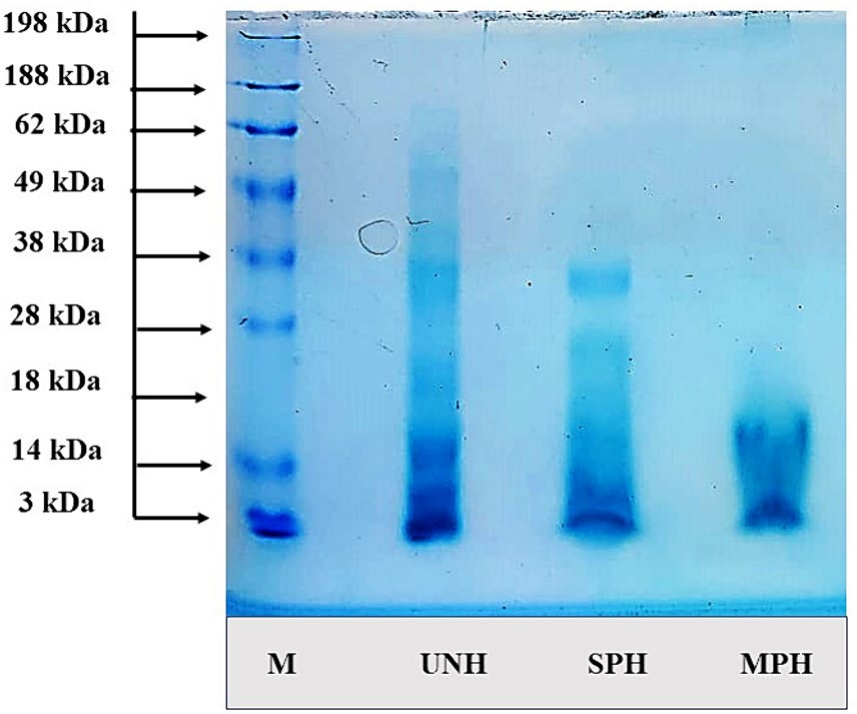
Molecular weight distribution of samples using SDS-PAGE. UNH, unhydrolysed soybean; SPH, soybean protein hydrolysate; MPH, modified protein hydrolysate.

The molecular weight (MW) distribution was illustrated using electrophoresis by SDS-PAGE (Figure 3). The MW of the SPH and MPH ranged between >3 kDa and 38 kDa. The bands in the gel showed that unhydrolysed soybean (UNH) showed bands up to 62 kDa indicating the high molecular proteins in it. On the other hand, SPH showed bands up to 28 kDa exhibiting a reduction in the molecular weight on hydrolysis. MPH showed thicker bands at 3 kDa and showed bands only up to 15 kDa. These results revealed that the modification of the hydrolysis process could efficiently decrease the molecular weights of MPH than SPH resulting in the formation of lower molecular weight peptides.

### 4.4 Physio-chemical characterization

#### 4.4.1 Amino acid composition of protein hydrolysates

Amino acid analysis was carried out to investigate the effect of the modified method on the residual amino acid contents of the sample. Amino acid compositions of SPH and MPH are given in Table 2. Among all the essential amino acids, arginine was found abundant in both SPH and MPH (19.90 and 11.79 mg/100 g, respectively). However, it was observed that there was a reduction in the amino acid content in MPH than SPH. This could be because of the degradation of amino acids during the hydrolysis process or thermal inactivation. Nevertheless, all the amino acid contents were found to be reduced in MPH. Tyrosine was absent in both SPH and MPH, whereas valine was found to be degraded in MPH.

**Table 2:** Amino acid content of soybean protein hydrolysate before and after method modification.

Sl. No	Component	SPH (mg/100 g)	MPH (mg/100 g)
1	Alanine	3.084	2.57
2	Arginine	19.90	11.79
3	Aspartic acid	1.48	1.54
4	Cystine	0.33	0.33
5	Glutamic acid	9.77	0.98
6	Glycine	0.74	0.72
7	Isoleucine	1.32	1.13
8	Leucine	9.86	6.61
9	Lysine	13.10	8.25
10	Methionine	1.73	1.11
11	Phenyl amine	11.54	9.88
12	Proline	7.28	5.95
13	Serine	1.13	1.13
14	Threonine	15.93	14.19
15	Tyrosine	ND	ND
16	Valine	3.65	ND

ND, not detected; SPH, soybean protein hydrolysate; MPH, modified protein hydrolysate.

#### 4.4.2 Particle size distribution of protein hydrolysates

Particle size analysis of protein hydrolysates demonstrates the structural dimensions of the powders. The percentage particle size of the samples is shown in Table 1. In the present study, the particle size of the UNH, SPH, and MPH ranged between 0.1 and 1,000 μm. It was observed that MPH had a higher of 28.62% of particles ranging between 0 and 100 μm, whereas SPH and UNH had 20.81 and 17.31%, respectively, which are statistically significant (*p* ≤ 0.05). Both SPH and MPH had 80% of the particle size within 500 μm, whereas UNH showed 69.9%.

#### 4.4.3 Water activity (a_w_) of protein hydrolysates

To determine the stability and shelf life of the dried samples from microbial and autolysis, a_w_ was analysed. In the present study, UNH, SPH, and MPH were analysed for their water activity (Table 1). The a_w_ of MPH was found to be significantly decreased compared to SPH and MPH (*p* ≤ 0.05), whereas a non-significant difference was observed between SPH and UNH. The a_w_ was found to be in the order of MPH < SPH < UNH.

#### 4.4.4 Color measurement of protein hydrolysates

The colour measurement was carried out to determine the hue and chroma of the hydrolysates. The results of colour measurements are shown in Table 1. L* values of UNH, SPH, and MPH showed 77.95, 73.83, and 72.99, respectively, whereas all the samples showed negative for a* and positive for b*. The results of L*, a*, and b* indicate the hydrolysates are light white with yellowish green in colour.

### 4.5 Evaluation of bitterness using e-tongue

The bitter taste in the protein hydrolysates is one major shortcoming to explore as it is a functional ingredient in nutraceutical food formulations. In the present study, the bitterness intensity of SPH and MPH was carried out using e-tongue. The bitterness concentration of the SPH and MPH is shown in Figure 2B. From the graph, it was observed that the bitterness concentration of SPH and MPH was found to be 0.48 and 0.12 mM/100 g, respectively. The results of this study revealed that the modification of soybean protein hydrolysates exhibited a 4-fold lesser bitterness concentration than the SPH.

### 4.6 Functional properties

#### 4.6.1 Oil-holding capacity (OHC) and solubility

**Figure 4:**
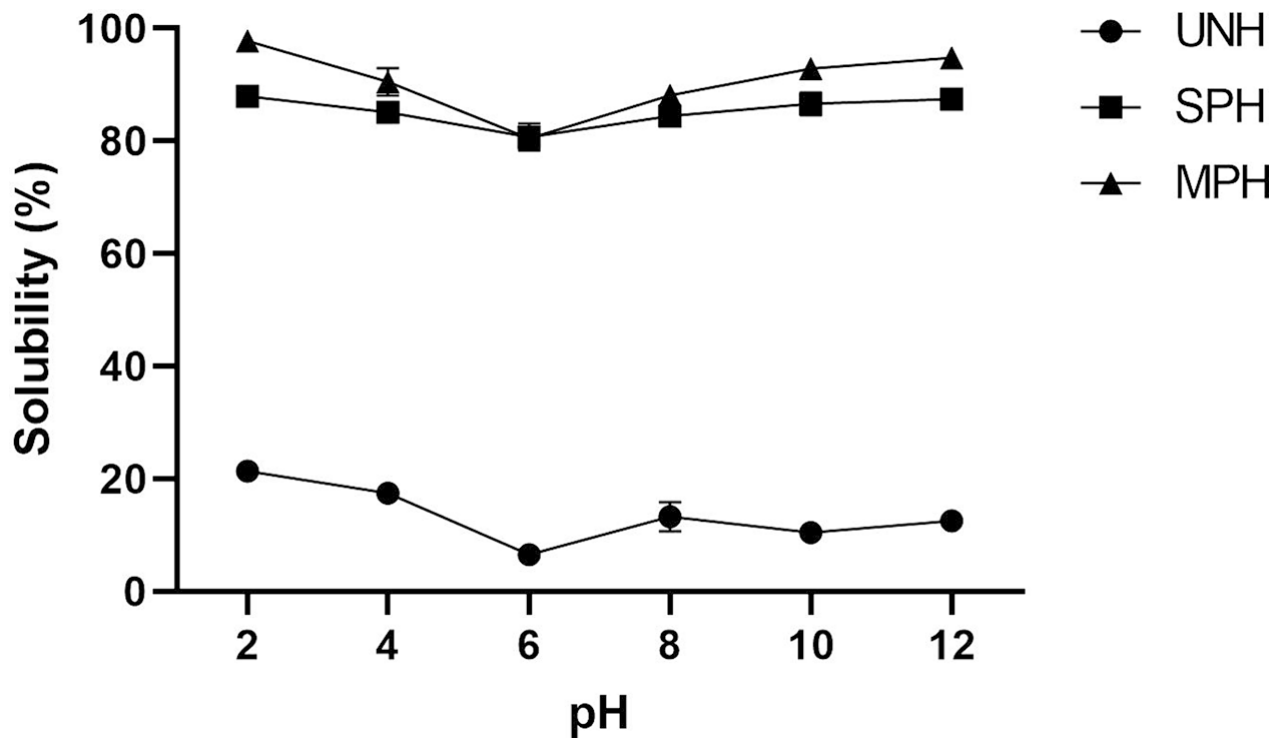
Solubility (%) of UNH, SPH, and MPH at different pH. UNH, unhydrolysed soybean; SPH, soybean protein hydrolysate; MPH, modified protein hydrolysate.

In the present study, the samples were evaluated for their OHC and solubility before and after hydrolysis. The OHC of all the samples is shown in Table 1. From the table, it was observed that the unhydrolysed soybean protein had higher OHC (4.74 g of oil/g), whereas SPH and MPH had 3.34 and 3.27 oil/g of hydrolysates, respectively. The OHC between SPH and MPH is statistically non-significant (*p* > 0.05). Solubility (%) in different pH (2–12) of the UNH, SPH, and MPH is shown in Figure 4. UNH showed a solubility (%) maximum of 21.41% at pH 2 and least at pH 6 (6.55%). SPH and MPH showed higher solubility (87.96 and 97.79%) at pH 2 and lower solubility (78.56 and 79.63%) at pH 6.

#### 4.6.2 Hardness of retrograded corn starch using texture profile analysis (TPA)

The effect of samples in reducing the hardness of retrograded corn starch was evaluated using texture profile analysis (TPA). The TPA of retrograded corn starch during the 0th and 7th day of storage is given in Table 3. The hardness of the gels incorporated with MPH showed significantly lower hardness than other samples. As observed in the table, the hardness of gelatinised corn starch (control) evidently increased (*p* ≤ 0.05) from 0.64 to 1.73 N during the course of storage from 0 to 7 days at 4°C, whereas the addition of 1% MPH showed an increase from 0.24 to 1.21 N. Nevertheless, other parameters such as fracturability, cohesiveness, and gumminess were also found to be altered by the addition of UNH, SPH, and MPH.

**Table 3:** Texture analysis parameters of retrograded corn starch during storage.

	**0**th **Day**					
	Parameters	CSC	CS + GG	CS + UNH	CS + SPH	CS + MPH
1	Hardness (*N*)	0.64 ± 0.01^a^	0.41 ± 0.02^b^	0.68 ± 0.009^c^	0.35 ± 0.009^d^	0.24 ± 0.24^e^
2	Hardness-2 (*N*)	0.44 ± 0.02^a^	0.38 ± 0.01^b^	0.49 ± 0.01^c^	0.29 ± 0.009^d^	0.25 ± 0.25^e^
3	Fracturability (*N*)	0.63 ± 0.0005^a^	0.05 ± 0.005^b^	0.08 ± 0.01^b^	0.21 ± 0.00^c^	0.05 ± 0.05^b^
4	Cohesiveness	0.38 ± 0.005^a^	0.51 ± 0.01^b^	0.68 ± 0.01^c^	0.42 ± 0.02^d^	0.27 ± 0.27^e^
5	Gumminess (*N*)	0.25 ± 0.008^a^	0.20 ± 0.005^b^	0.47 ± 0.01^c^	0.15 ± 0.02^d^	0.07 ± 0.07^e^
6	Chewiness index (*N*)	0.18 ± 0.01^a^	0.11 ± 0.01^b^	0.41 ± 0.005^c^	0.11 ± 0.01^b^	0.02 ± 0.02^e^

	7th Day					
1	Hardness (*N*)	1.73 ± 0.02^a^	2.17 ± 0.005^b^	1.82 ± 0.02^c^	1.55 ± 0.04^d^	1.20 ± 0.005^e^
2	Hardness-2 (*N*)	1.52 ± 0.03^a^	1.71 ± 0.02^b^	1.06 ± 0.00^c^	0.65 ± 0.01^d^	1.14 ± 0.05^e^
3	Fracturability (*N*)	0.26 ± 0.04^a^	2.18 ± 0.02^b^	0.37 ± 0.02^c^	0.41 ± 0.01^c^	0.17 ± 0.02^d^
4	Cohesiveness	0.4 ± 0.009^a^	0.5 ± 0.02^b^	0.37 ± 0.01^a^	0.42 ± 0.02^a^	0.43 ± 0.02^a^
5	Gumminess (*N*)	0.67 ± 0.02^a^	1.1 ± 0.00^b^	0.69 ± 0.01^c^	0.62 ± 0.02^a^	0.53 ± 0.02^d^
6	Chewiness index (*N*)	0.49 ± 0.00^a^	1.13 ± 0.04^b^	0.42 ± 0.01^c^	0.6 ± 0.02^d^	0.17 ± 0.01^e^

Values are represented as triplicates of mean ± SD. The mean value with different alphabets in the superscript between the columns in the same group indicates a significant difference (*p* ≤ 0.05) determined using two-way ANOVA. CSC, corn starch (Control); CS + GG, corn starch + gaur gum; CS + UNH, corn starch + unhydrolysed soybean protein; CS + SPH, corn starch + soya protein hydrolysate; CS + MPH, corn starch + modified protein hydrolysate.

### 4.7 Effect of modification of soybean hydrolysis on protein digestibility

**Figure 5:**
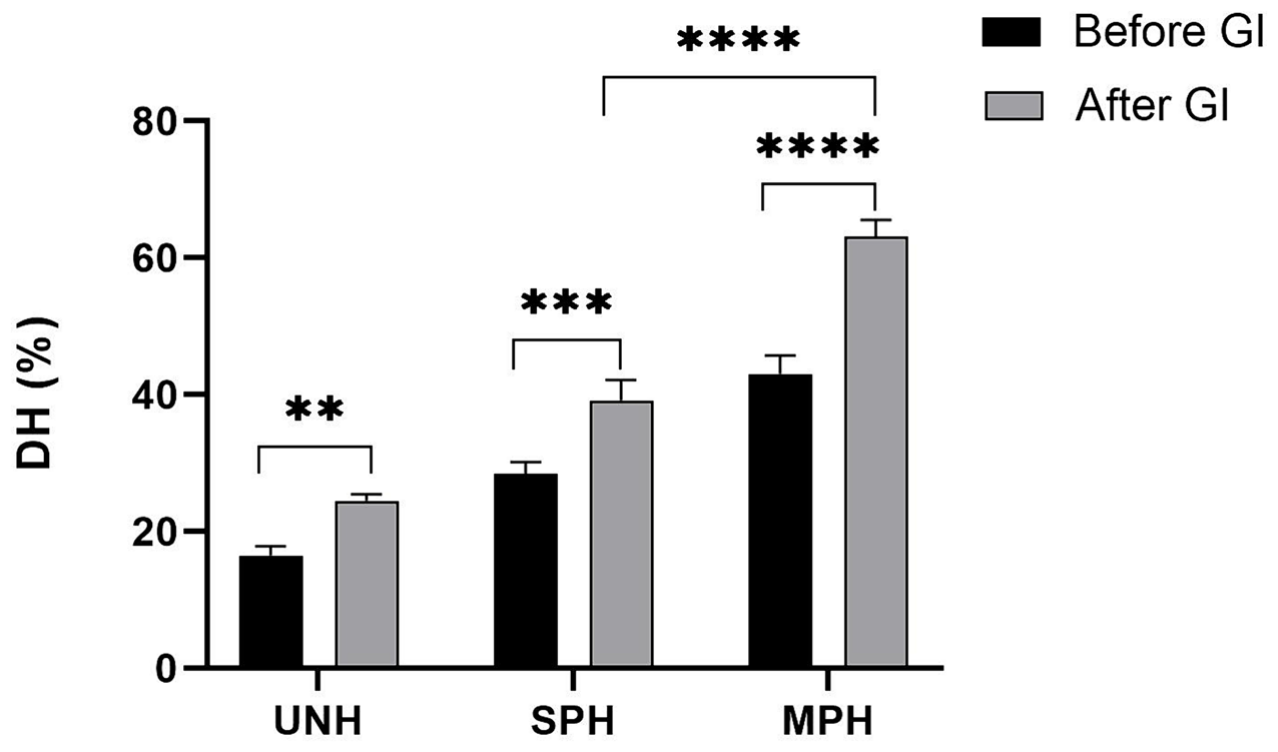
Effect of gastrointestinal digestion on the degree of hydrolysis of UNH, SPH, and MPH. UNH, unhydrolysed soybean; SPH, soybean protein hydrolysate; MPH, modified protein hydrolysate. *Statistical significance at *p* ≤ 0.05. **Statistical significance at *p* ≤ 0.01. ***Statistical significance at *p* ≤ 0.001. ****Statistical significance at *p* ≤ 0.0001.

In the present study, the protein digestibility of all the samples was determined by GI digestion followed by estimating the DH% (Figure 5). The results showed that the protein digestibility was found to be higher in MPH than in SPH (*p* < 0.05). All the samples showed significantly increased DH% upon GI digestion. On the other hand, GI-digested MPH showed 1.61-fold higher DH than GI-digested SPH and 2.57-fold higher than UNH. These findings illustrate the importance of modification on increased protein digestibility of MPH than others.

### 4.8 Safety evaluation of SPH and MPH

**Figure 6:**
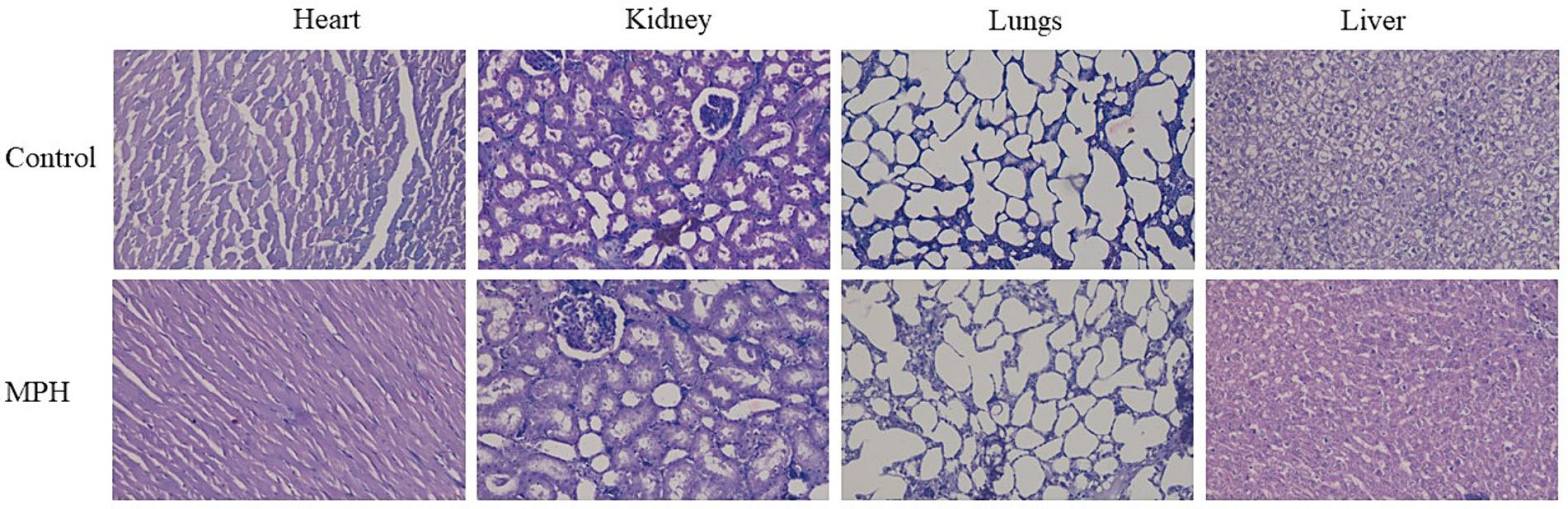
Histopathological examination of heart and lungs of Wistar rats dosed with MPH at 2000 mg/kg B.W. MPH, modified soybean protein hydrolysate.

The SPH and MPH were assessed for their cell proliferation ability using RAW264.7 macrophage cell lines. The cell proliferation was found to be 115 and 118% in SPH and MPH, respectively. The results indicate that both SPH and MPH have higher LC_50_ concentrations and are likely to be non-toxic. This result was further confirmed by *in vivo* acute toxicity assay. The *in vivo* toxicity using male Wistar rats was assessed to examine any behavioural, neurological, and histological changes. No changes were observed in the neurological functions or behaviours such as aggressiveness, alertness, touch response, and motor activity, following the oral administration of MPH. The histopathological observation of vital organs using eosin staining is shown in Figure 6. From the histopathology results, it was found that there were no changes in the function as per the histological study.

## 5 Discussion

### 5.1 Physicochemical properties of soybean protein hydrolysate before and after modification

Protein hydrolysates are comprised of active peptides and exhibit therapeutic potential against lifestyle disorders (27). Scientific investigations underscore their bioactive properties and enhanced functional attributes (4, 5, 28–30). Despite their promising role in nutraceutical foods, their practical application is currently constrained primarily by production costs, alterations during digestion, and suboptimal palatability (2, 8). Reports indicate that protein hydrolysates with higher DH% and peptides of lower MW often exhibit favourable functional and bioactive properties (4, 31, 32). Our study focussed to enhance the palatability and functional characteristics of protein hydrolysates through modified hydrolysis processes, specifically by adjusting time and enzyme concentration in two phases. The study aims to reduce the bitterness and improve the functional properties of soya protein hydrolysate.

Proteolytic enzymes play a vital role in generating protein hydrolysates, with the DH% indicating the extent of the enzyme-substrate reaction (33). The increased DH% in MPH is likely due to hydrolysed oligo-peptides from Phase-I undergoing further hydrolysis into smaller peptides in Phase-II, without interference from unhydrolysed native proteins. Alcalase is known for its broad substrate specificity, which is an endo-peptidase that cleaves hydrophobic amino acids such as tryptophan, phenylalanine, leucine, isoleucine, valine, and methionine residues (34). Yathisha et al. (4) reported that hydrolysis with alcalase yielded a higher DH (38%) in ribbon fish protein hydrolysates compared to other enzymes such as flavourzyme and papain (12 and 18%, respectively). The MW distribution of protein showed bands ranging between 3 and 15 kDa in the MPH sample, while SPH and UNH exhibited wider ranges. Similar changes in MW were observed in Sacha inchi (*Plukenetia volubilis*) protein hydrolysates reported by Rawdkuen et al. (35) using papain and Calotropis proteases. Suresh (36) noted an increase in bands in the lower MW region with higher enzyme concentration and hydrolysis time. These results are also supported by the particle size of the SPH and MPH which showed that 78.4% of the particles have 0–500 μm size. Studies have reported the fact that protein hydrolysate showing lower MW peptides possesses higher functional and bioactive properties (2, 16, 37).

The development of bitterness in the hydrolysis of protein is a major challenge in food industries to be used as a functional ingredient (7). According to Fan et al. (38), the presence of hydrophobic amino acids in the protein hydrolysates, such as phenylalanine, proline, leucine, arginine, valine, lysine, and histidine, can be attributed to the bitterness. The author also states that hydrolysis with alkaline protease produced hydrolysates with the highest bitterness. In our study, modification of protein hydrolysate preparation using alcalase exhibited effectiveness in reducing the bitterness, showing 4-fold lesser than SPH. Correspondingly, MPH showed reduced hydrophobic amino acids such as proline, phenylalanine, leucine, isoleucine, and complete destruction of valine when compared to SPH. These findings corroborate with the results reported by Newman et al. (39) and Fan et al. (38). The author states that prolonging hydrolysis time beyond a specific point results in a decrease in bitterness due to the eventual hydrolysis of the bitter peptides. Changing the proteolytic enzymes can also reduce the bitterness in the hydrolysates (11). Studies have also reported that other treatments with activated carbon, extraction with alcohol, use of cyclodextrins, chromatographic separation, hydrolysing further with the exopeptidase, and use of butanol can also aid in de-bittering (8, 10). Another study by Zhang et al. (7) stated that soya protein hydrolysates with alcalase and cross-linking with trans-glutaminase (TGase) could reduce the bitter-active peptides lowering bitterness and improving the overall flavour. However, these methods are also reported to affect protein hydrolysate yield, altering the structure and bioactive properties.

### 5.2 Effect of modification on functional properties and protein digestibility of soybean protein hydrolysates

Functional properties are the physio-chemical properties of proteins that influence their behavior during processing, handling, and consumption. Hydrolysis of protein using proteolytic enzymes produces peptides of different chain lengths and free amino acids which modifies the properties of native proteins such as solubility, oil-holding capacity, swelling capacity, foaming, and emulsifying capacities. Generally, protein hydrolysates with higher DH increase the ionisation of amino and carboxyl side chains of amino acid residues increasing the hydrophilicity. Hydrolysis develops interactions between the hydrophobic peptides and the remaining intact protein in hydrolysates causing a decrease in hydrophobicity of the protein surface, thereby increasing their solubility (40). Similar results were observed, where UNH had the least water solubility at different pH ranges. Conversely, SPH and MPH showed higher solubility in the acid and alkaline media whereas solubility was found to be decreased in the low acidic to neutral pH. The decrease in solubility of approximately 5.0–6.0 pH is due to the general isoelectric point for seed proteins (41). The solubility of walnut protein hydrolysates was found to be increased as the hydrolysis time increased (42). These results were consistent with the findings of Mokni Ghribi et al. (43) in chickpea protein hydrolysates using alcalase. Horax et al. (40) reported maximum solubility (74.5% at pH 3) of soybean protein hydrolysates (40). In the present study, the OHC of the samples was observed to decrease accordingly as the DH% increased. However, the OHC of SPH and MPH was observed to be non-significant (*p* ≤ 0.05). These findings are similar to the study carried out with cherry kernel protein concentrate and hydrolysates by Cingöz and Yildirim (44). The author reports that the OHC of the hydrolysates is not solely dependent on the hydrolysis or DH but may be due to the hydrolysis process which might reduce the oil-trapping surfaces. Similar results were also reported by Guan et al. (45) in which the OHC was found to be decreased in the trypsin hydrolysed oat bran protein compared to untreated protein. These findings also throw light on the modification of hydrolysis in improving the functional properties over the traditional process. Retrogradation is a phenomenon in which the gelatinised starch hardens over a period of time by the re-association of amylose and amylopectin removing water molecules from it (13). In our study, the influence of MPH on the hardness of gelatinised starch showed a significant reduction in hardness even after storing for 7 days at 4°C. Our results also correlate with the findings of Hu et al. (12) and Luo et al. (13), in which addition of whey protein and grass carp protein hydrolysates decreased the hardness of the gelatinised rice starch. In the present study, the MPH showed a higher tendency to reduce the hardness of the corn starch over SPH and UNH. The inhibition of the formation of hydrogen bonds among the starch chain is because of the interaction between the smaller peptides with the starch molecules and their formation of the starch–protein complex (13). These findings also support our findings of decreased hardness of retrograded starch.

Protein digestibility is one of the important characteristics which determines the bioavailability of protein hydrolysates. In the present study, the protein digestibility of all the samples was assessed by GI digestion followed by estimating the DH%. The increase in the DH% indicates a large number of peptide bonds being cleaved during GI digestion, which implies an increase in protein digestibility (46). The author also reported that modifications in the protein structures during processing (hydrolysis) may expose more peptide bonds for cleaving during GI digestion (47). Our results also corroborate with these studies as the protein digestibility was found to be in the order of MPH > SPH > UNH. Studies also suggest that lower molecular weight peptides are more likely to be absorbed in the body than high molecular weight peptides. Our previous review also supports the fact that GI digestion can alter the molecular weight of the peptides and have both positive and negative effects on absorption and bioactive properties (2, 37). Studies have also proven that increased DH% shows higher bioactive and functional properties (4, 16, 48). Hence, the increased DH% after GI digestion can improve the bioaccessibility of protein for absorption in the intestine. On the other hand, the effect of GI digestion on the bioactive properties needs to be studied to depict its efficiency on the bioactive properties of MPH. Hence, the use of MPH as a high protein constituent makes them a prominent food additive. The influence of protein hydrolysates on high-calorie food can be investigated to understand their functional attributes. Liu et al. (49) studied the suitability of soy protein hydrolysates as a fat replacer in ice cream. These findings also provide additional application of MPH in promoting its use as a food additive for food processing industries.

### 5.3 Safety evaluation of soybean protein hydrolysates

SPH and MPH exhibited higher cell proliferation in normal cell lines. This could be because the essential amino acids in both SPH and MPH would promote cell growth by increasing the cell metabolic activity in the test group. Similar results were also obtained in the *in vivo* toxicity assay in which the test group rats did not show any behavioural changes. The histopathological examination also proved that there were no significant pathological changes among the test group and control group. This might be because of the presence of essential amino acids in SPH and MPH that could promote cell growth in *in vitro* and harmless effects in *in vivo*. Other studies have also suggested that protein hydrolysates are likely to be non-toxic and can be consumed without causing any major side effects (24, 26). According to Umayaparvathi et al. (50), protein hydrolysates and isolated peptides exhibit cytotoxicity in cancer cells, but on the other hand, they are also non-toxic in normal cell lines. This could be because of the selective cell proliferation behaviour of the bioactive compounds.

## 6 Conclusion

The study aimed to investigate the functional properties, palatability, and protein digestibility of MPH over SPH and UNH. Despite various methods employed for de-bittering protein hydrolysates, it often incurs high costs, reduced yield, and alterations in properties. Contrary to these challenges, our study demonstrated that MPH maintained functional group stability, enhanced functional properties, and reduced bitterness intensity without any additional process. The outcomes of the study emphasise the promising functional properties of MPH and its potential in the food and pharmaceutical industries, providing valuable insights for improving properties with reduced bitterness. The cause of high functionality and decreased bitterness needs to be studied in detail. Furthermore, the findings can have applications in the broader food industry, potentially influencing the development of new and improved food formulations.

## Data Availability

The raw data supporting the conclusions of this article will be made available by the authors, without undue reservation.
